# Grasping the Situation: analyzing how situational dynamics shape agency

**DOI:** 10.3389/fpsyg.2024.1392995

**Published:** 2024-07-23

**Authors:** Thijs Heijmeskamp

**Affiliations:** ^1^Erasmus School of Philosophy, Erasmus University Rotterdam, Rotterdam, Netherlands; ^2^Faculty of Industrial Design Engineering, Delft University of Technology, Delft, Netherlands

**Keywords:** situated agency, situated cognition, affordance, emotion, sociomateriality, John Dewey, situation, affect

## Abstract

Despite the intimacy between the situation and our agency, “situation” remains an ambiguous concept in theory. Even within the context of situated theories of cognition and agency that take the organism-environment system as central in their investigations, the notion of “situation” has been undertheorized. Yet, whether affordances are relevant depends on the situation. Therefore, Van Dijk and Rietveld argue that we must understand the practical situation in which behavior occurs in order to know how we respond to the affordances that the materials and other people offer. Taking John Dewey’s notion of “situation” as the basis for investigation, I follow Shaun Gallagher’s analysis of how we are not just part of a situation, but we understand what an action is only in relation to a situation. Situations act like large-scale affordances, but this does not mean that affordances are inviting or soliciting as such. Because of the situational transactions with the environment that an agent has, the environment pushes and pulls the agent from and toward certain actions. This means that environments have expressive qualitative features that are non-subjective emotional qualities and social gestalt. I propose four overlapping but distinct features or axes of analysis of situations that can be identified and analyzed in terms of how they shape our agency: complexity, determinedness, the establishment of expectations, and restrictiveness. Situations can be more or less complex in a spatial, temporal, or layered way. They can also be more or less determined, meaning that the agent’s actions are more or less obvious. Third, they can be characterized as socially established, meaning that certain behavior is expected. Finally, situations are more or less restricted, denoting the number of activities available to an agent.

## 1 Introduction

We “find ourselves in situations,” sometimes we “have to face a certain situation,” and sometimes “the situation forces our hands.” Referring to a situation is often enough to explain our actions. A situation is something that falls upon us, yet at the same time, something that we can help create. Situations can be small, such as having a Sunday breakfast at home. Situations can be enormous, such as finding yourself in a war. Despite the intimacy between the situation and our agency, “situation” remains an ambiguous concept in theory.^1^ Even within the context of situated theories of cognition and agency that take the organism-environment system as central in their investigations, the notion of “situation” has been undertheorized (e.g., McGann, 2014).^2^ Yet, a situation is powerful in shaping our agency as we must adapt to its demands. For instance, although a chair might afford me to sit down, I would not act on the affordance, knowing the seat was already taken by someone else. An alleyway might afford to walk through it, but I still avoid it if it is unlit in the dark. Although the affordances are there, they might not be *relevant* in the current situation. van Dijk and Rietveld (2017, p. 2) point out that affordances are situated, and in order “to understand how we respond to affordances offered both by material aspects of the environment and by other people, it is crucial that we understand the practical situation in which such behavior occurs.” That is, I argue, that the notion of “situation” best describes the practical conditions that shape our agency.

Taking John Dewey’s notion of “situation” as the basis for investigation, I follow Gallagher’s (2020) analysis of how we are not just part of a situation, but we understand what an action is only in relation to a situation (Section 1). I clarify this relationship between action and situation through van Dijk and Rietveld’s (2021) distinction between activity and action, where an action is what activity turns out to have accomplished, and activity is the process of accomplishing an action. Situations act as large-scale affordances that structure the activities’ relevant affordances underlying the action (Section 2). However, situations that act like large-scale affordances do not mean that affordances are inviting or soliciting as such.^3^ Because of an agent’s situational transactions, the environment pushes and pulls the agent from and toward actions. I argue that this means that environments have expressive qualitative features that are non-subjective emotional qualities and social gestalt, adding a new twist to what Matthew Crippen (e.g., Crippen, 2021, 2022) has asserted (Section 3). A mountain becomes less climbable if we are tired or depressed. These are not properties that the agent projects on the environment but are part of the environment in relation to the agent. Ironically, the notion that environments have a qualitative character is a notion that Gibson (1979, pp. 139–40) rejected in his critique of the notion of “demand character” in Gestalt psychology. The environment, which has to have a qualitative character, can also be found in Dewey. We perceive the environment not just as containing affordances but affordances that push and pull us in particular ways. We (almost) never have a neutral relationship with our surroundings because our relationship with the environment is effectively mediated. Crippen (2022) points out that because humans are fundamentally social creatures, they create and perceive emotionally inflected spaces (Section 4). Therefore, I argue that this means that situations turn spaces into a setting for action and into territories for certain forms of agency. Many spaces are habitually marked for certain forms of agencies and are territories.

Because the situation centers on the interaction between agent and environment, we gain more tools for analyzing the situated nature of agency, besides the agential and environmental dynamics. I end by proposing four overlapping but distinct features of a situation that can be analyzed in terms of how they shape our agency. First, a complex situation allows for a more complex series of actions over time. Second, following Dewey, an indeterminate or problematic situation blocks action until the indeterminacy is resolved. Third, a situation can also be well-established in the sense that social coordination becomes easy. Situations not only shape expectations for ourselves but also for others. In those cases, referring to the situation in question is perfectly acceptable as an explanation for behavior. Fourth, a situation can be too restrictive. This means that a situation limits the possible meaningful interactions with the environment and even prevents the agent from establishing new meaningful interactions.

To understand action and its context, we must get a grasp on what a situation is. Viewing context as environment means talking about the organism-environment relation in terms of the organism’s capacities and how those relate to the world. To view context as a situation is to conceptualize the organism in terms of acting and transacting with its environment. Both conceptualizations of context are needed for our understanding of agency. Despite the richness of theories of embodiment and situated agency and cognition, very little has been written about what a situation entails, even by those that take the organism-environment system as the main unit of analysis. The notion of situation allows us to conceptualize how agency, including moral and political, is environmentally scaffolded without losing the active part of the agent nor its fundamental situatedness.

## 2 Dewey’s notion of situation

We live in worlds filled with countless options for action. The chair I am currently sitting on affords me the option of sitting. However, I could also use it as something to stand on to get to something that is out of reach, to change a light bulb for instance. Just like Gibson’s rock (Gibson, 1979, p. 134), which is both a missile and a paperweight, a chair, like most objects, affords multiple actions. However, this does not mean that all affordances are equally salient. What holds for chairs also holds for spaces. A typical space, like an office room, holds even more numerous possibilities for action. Yet, it is only rarely that we are overwhelmed with indecision. Even if we think about what to do, most possibilities are not considered. At the same time, spaces can be very constraining or force us toward certain actions and limit our agency.

The answer to the problem of “when does an action become relevant?” is usually that agents have interests and/or goals that determine our actions when approaching an object or space. However, at best, this merely shifts the problem to what goals and interests are relevant in a given situation. I do not walk into a room, perceive the chair as an object among all the other objects, and then go through the list of interests and goals in my head to think about what applies the most. Wu (2011, 2016) defines the problem that we are surrounded by multiple possibilities for action, the *Many–Many* problem: an agent is confronted with (too) many perceptual inputs and (too) many possible behavioral outputs. According to Wu (2011, p. 53), it is the context of the action that “typically presents an agent with this many–many manifold that delineates a behavioral space of possible actions at a time.” The problem holds if we reformulate it in terms of affordances: in typical cases, the agent is surrounded by too many affordances that the environment offers. The proposed solution is often framed as some affordances are more soliciting or inviting than others (e.g., Withagen et al., 2012; de Haan et al., 2013; Rietveld and Kiverstein, 2014). However, what makes an affordance soliciting or inviting remains unclear, as the soliciting or inviting nature of the affordance depends on environmental and organismal factors (Withagen et al., 2012), i.e., the context of action. Affordances are situated and, therefore, an appeal to context or situation must be made to complete the explanation of how we arrive at performing a certain action. Yet, despite an appeal to context, what such a relation entails remains unclear.

We can begin by clarifying this relation between action and context by looking at how we contextually understand our actions. Shaun Gallagher argued that the context of action could best be conceptualized through the notion of “situation” and approached the relation between action and context through an example originally provided by Elizabeth Anscombe:^4^

“[A] single action can have many different descriptions …. Are we to say that the man who (intentionally) [A] moves his arms. [B] operates the pump, [C] replenishes the water supply, [D] poisons the inhabitants, is performing four actions? Or only one?…” (Anscombe, as quoted by Gallagher, 2020, p. 7).

“Moving the arm up and down on the pump handle” being the relevant description of action entails different circumstances than “poisoning the household.” The former requires at least a context where the agent was not aware they were poisoning the household. Separating A, B, C, and D, although they may be instrumentally related, such as A and B, into two actions seems like an abstraction that ignores circumstances. Describing A, B, C, and D as multiple actions results in excluding context. For instance, if the agent is aware that they are poisoning the household, and A, B, and C plus certain contextual elements dynamically constitute poisoning the household, then the contextually relevant description of pumping the water can be poisoning the household. What the relevant description of action is depends on the circumstances. Gallagher (2020, p. 9) argues that the descriptions A, B, C, and D describe four different sets of contexts of one action if we take context to include both agent-related aspects, such as intention, motivation, skill, and knowledge, and world-related aspects, such as facts about objects and physical arrangement. Since these are all aspects of one action, they contribute to what is *relevant* from the perspective of an agent and the perspective of an interested observer. A police officer looks at this example differently than a kinesiologist. The former is more interested in the murder, while the latter is more interested in a description of the action in terms of muscle and movements of the joints. Usually, we are not interested in the latter as a means of interpreting the actions of others or in other abstract terms such as beliefs, but we make sense of the “actions of others in terms of their goals and intentions set in contextualized situations”^5^ (Gallagher, 2020, p. 107). The same holds for the object that is manipulated in the action. To borrow an example from James (1879, p. 319), oil is both a combustible, a lubricant, and a wood darkener, depending on the needs of the user. In other words, we understand the actions of others and ourselves at the most *relevant* pragmatic (intentional and goal-oriented) level and generally ignore lower-level descriptions (Gallagher, 2020, p. 107). The relationship between action and context also holds for the agent. For instance, the person pumping water from the well is getting water for the household, but under other circumstances, it might be poisoning the household, such as the person knowingly trying to kill the dictator that lives there (Anscombe, 2000, p. 37). Both getting the water and poisoning are aspects of one action that can be described differently depending on the context, which involves both an agent side and a worldly side.

Gallagher argues that the context of action, without losing the idea that context involves both an agent side and a worldly side, is best characterized through the notion of situation as developed by John Dewey (Gallagher, 2020, p. 13). Context, in the form of a situation, is part of what constitutes an action. In Dewey’s 1938 *Logic*, we find the following definition of situation:

“What is designated by the word “situation” is not a single object or event or set of objects and events. **For we never experience nor form judgments about objects and events in isolation, but only in connection with a contextual whole**. This latter is what is called a “situation.” […] In actual experience, there is never any such isolated singular object or event; an object or event is always a special part, phase, or aspect, of **an environing experienced world—a situation**. The singular object stands out conspicuously because of its especially focal and crucial position at a given time in determination of some problem of use or enjoyment which the **total complex environment presents. There is always a field in which observation of this or that object or event occurs**. Observation of the latter is made for the sake of finding out what that field is with reference to some active adaptive response to be made in carrying forward a course of behavior.” (Dewey, 1986, pp. 72–73, emphasis mine)

Dewey defines a situation as an environing experienced world. It is experienced, because for Dewey (2013), experience is made up of all our transactions with the world. It is also environing, that is, we experience objects and events against a meaningful backdrop of a contextual whole. A situation is not a single isolated experience like stubbing my toe on the coffee table. Situation, as a particular aspect of context, is our primary means of relating to and understanding the world. The claim, therefore, is that we perceive a scene or setting, just like we do not see singular objects as the sum of their properties. In a letter to a friend, Dewey expands on the notion of situation:

““Situation” stands for something inclusive of a large number of diverse elements existing across wide areas of space and long periods of time, but which, nevertheless, have their own unity. This discussion which we are here and now carrying on is precisely part of a situation. Your letter to me and what I am writing in response are evidently parts of that to which I have given the name “situation”; while these items are conspicuous features of the situation they are far from being the only or even the chief ones.” (Dewey and Bentley, 1949, p. 315)

According to Dewey, we experience the world through situations as they are the background on which action takes place, and action makes sense to us. What determines the limits of a situation is the *relevance* of things, events, processes, etc., to the action or activity. A situation is not equivalent to an umwelt or an environment, as the organism is not in the situation but a part of the situation. The situation is constituted by the relational nature of the organism-environment, which means that the situation includes the agent or experiencing subject. We are situated in an environment, but our countless transactions with the environment have a unique situation each that the transaction is part of Dewey (1997, p. 25), who already theorized about the centrality of the situation in experience, states that “[t]he conceptions of situation and of interaction are inseparable from each other.”^6^ To live in a situation is to be in a transaction, and living in a world means living in a series of situations. Therefore, an agent cannot step outside of a situation without changing it, nor can an agent point to a situation as the pointing is part of the situation (Gallagher, 2020).

Dewey anticipated, for instance, enactivism and ecological psychology in that we do not respond to stimuli of particular objects in the environment but relate to the elements of a meaningful environment as a whole to which we respond in a certain manner, making certain objects salient. Perception is the coordination of the whole organism, with the body as its mediator. We experience most things as a mixture of our senses, and we generally perceive in terms of action because perception is a sensorimotor coordination, in that perception and action (or stimulus–response) are not separable as each perception is a sensorimotor coordination by itself (Dewey, 1896, p. 366). For instance, the feeling of smoothness of the table can only come about by us moving our fingers over the surface of the table. Because of the role of the body, the overall perceptual situation affects how we experience details of that context: “A waterfall that sounds pleasant in the context of a park becomes irritating when recorded and played out of context and is sometimes mistaken for traffic” (Crippen and Schulkin, 2020, p. 80).

A situation is defined in relation to the performance of action (Gallagher, 2020, p. 13). It contains activities, an active organism, and environmental objects that delineate possibilities for action. For me, writing this article, the situation contains a desk, a laptop on the desk, and a desk chair as its *conspicuous* elements. The situation also contains colleagues working around me. If someone were to remove my desk, I could sit in that space but not lean my elbows on where the desk was. The arrangement and the rearrangement of (objects in) the space is a rearrangement of the situation. However, the exact space is a completely different situation for a cat because a cat has wildly different capacities (Crippen and Schulkin, 2020, p. 36). For the cat, the seat of the chair that sits under the desks affords an excellent sleeping spot. Because of our different capacities, that is, differences in bodily organization such as skills, we are confronted with different constraints and possibilities; I perceive the situation as different than my cat, meaning that we are in objectively different situations. “Situations are where things first exist and appear to us” (Crippen and Schulkin, 2020, p. 66). Actions take place at the level of a situation. It is through situations that we interact with a meaningful environment. Or the other way around, we have an environment because we relate to the world through situations. A situation is a relation of relevancy between elements of the environment given certain practices and activities.

## 3 Situation as affordance-like

In Anscombe’s example of the water pump, if the action was poisoning the household, the action consists of many activities, such as the activities of pumping the water, bringing the water to the other members of the household, and all the activities associated with being a political agent. The performance of activities is something that unfolds over time and needs to be sustained by the effort and skill of those performing the activity. There are many activities nestled in other activities, such as pumping up the water, which consists of many related motor activities. Although the intention of an agent is important for the context and how we could describe an action, the intention does not determine which activities are part of the overall action (Gallagher, 2020, p. 12). We usually adapt quite easily to the demands of the situation as it unfolds. Action, activity, and situation have a temporal structure. Examining this temporal structure allows us to consider how a situation organizes activities and acts as an affordance itself.

Experience of activities and experience as such has a temporal structure in that it has a retrospective and prospective quality. It is retrospective, as James (1950, p. 240) argued that “what we hear when the thunder crashes is not thunder pure, but thunder-breaking-upon-silence-and-contrasting-with-it.” Not only is the current activity constrained by material conditions, but prior activities, decisions, and contexts have led to the activity and constrain how it is experienced. For example, the difference between experiencing drinking water after a long walk in the desert versus the experience of drinking water on a normal day at home. Our experience of activities is prospective, as sensorimotor coordination has an anticipation of certain conditions of fulfillment. We anticipate that drinking water will quench our thirst. Just like our experiences of particular things and affordances are nested in our experience of other things and their affordances, so are activities nested in other activities. Action and activity are one and the same process, and the distinction is temporal, in that action is backward-looking and activity is forward-looking. Action is what activity accomplishes, and activity is the accomplishing of action (Schatzki, 2012, p. 19; van Dijk and Rietveld, 2021, p. 355). Van Dijk and Rietveld argue that:

“Any activity that appears as a finished *action* can be part of a continuing string of actions that forms a larger *activity* still unfolding. Like a key-press, activities are often nested in other activities.” (Van Dijk and Rietveld, 2021, p. 8)

Activity is ongoing and open for continuation, while the action becomes more determinate. When my hands move to the keyboard of my computer to press a certain key, the available activities decrease as my fingertips approach certain keys. Rietveld and Van Dijk point out that action, as a temporal phenomenon, has a fundamental indeterminacy, and the action is only completely determined once the activity is over. As the action becomes more determined, the available activities decrease. Possible ways of continuing activity make way for the actual manner in which activity is continued. This also means that certain activities become relevant to continue or engage based on their relation to the overall unfolding situation. Thus, while an activity is performed, a specific way of doing and coordination of materiality unfolds that constitutes an action.

Van Dijk and Rietveld (2021, p. 358) argue that the same temporal relationship between action and activity also holds for affordance. Furthermore, a process view of activity implies a process view of affordance (van Dijk and Rietveld, 2021, pp. 355–56). The basis of such a process view can be found in Rietveld and Kiverstein’s understanding of affordance.^7^ Building on what Gibson (1950, 1979) states and expanding on sides of his study that Chemero (2003) especially highlights, Rietveld and Kiverstein argue that “affordances are relations between aspects of a material environment and abilities available to a form of life” (2014, p. 335). Affordances are relational because they depend on the skills the agent possesses. However, for an individual, an affordance is also a resource. Affordances are relational with respect to a form of life. If an individual has the relevant abilities, they can pick up on an affordance (Rietveld and Kiverstein, 2014, p. 340). The form of life of an animal is its relatively stable and regular ways of doing things. An affordance is not merely material but often also sociocultural. Our actions and practices are not merely constrained by physical factors but also by social coordination, as objects are part of a variety of (social) practices. For instance, a chair is used differently when it is part of the game Musical Chairs than when used to sit on for work. Van Dijk and Rietveld (2017) conclude that the relational nature of affordance means that our experience depends on the arrangements of a sociomaterial environment. Sociomaterial means that social coordination cannot be separated from the material object (cf., Mol, 2002). Because we relate to the environment not only as a contextual whole spatially but also from a history of previous interactions or practices, we are attuned to patterns in the environment that invite us to respond in certain ways. For instance, a lot of activities at home are anchored in the dinner table. We can lean on the dinner table, and the dinner table offers the possibility of sitting down with others, sharing a meal, and discussing how the day went or is going to be. These actions are not just possible because of the physical affordance but also the social coordination that is anchored in the artifact.

According to van Dijk and Rietveld (2021, p. 362), an affordance can invite activities, and by doing so, it sets up its conditions for its own continuation, maintenance, and development. Furthermore, the notion of affordance is scalable. Activities and actions can take on a larger significance as they are part of an overarching activity or action. Affordances at shorter timescales can intertwine and constitute a different affordance unfolding at a larger timescale. For an agent, this usually means knowing what to do. Skilled individuals who have the relevant ability, “have acquired the responsiveness to attune to the direction of unfolding affordances along such larger timescales” (van Dijk and Withagen, 2016; van Dijk and Rietveld, 2021, p. 362). Because they are able to follow along, these individuals can be invited by affordance at the larger timescale to participate and make it unfold further (Heft, 2001; van Dijk and Rietveld, 2021). It is not necessary for an individual to know all the steps of the process or have a specific picture of the very end of all the activities to be invited and engaged. A large-scale process can keep unfolding as long as it invites the agents participating in the process to enact the relevant smaller-scale affordances. The process of building a wall makes picking up and placing down bricks in a certain position relevant, thereby allowing the agent to eventually plaster and paint the wall. In a situation, we anticipate certain actions through affordances, and that guides our activities and, therefore, the unfolding of a situation. Thus, a situation temporally constrains activities, making some relevant for the continuation of action.

Van Dijk and Rietveld (2021, p. 359) argue that an activity following up on a previous activity or action makes up a large-scale activity that is also being enacted. We can anticipate the unfolding of action because we have anticipatory responsiveness to a ‘large-scale’ affordance and thereby anticipate situations and possible actions to unfold in the situation. Van Dijk and Rietveld provide the making of an architectural art installation as an example and case for analysis of a large-scale affordance. The architects saw the affordance despite the installation not yet existing or planned out, as the activities of making the installation were exploratory to create something novel (van Dijk and Rietveld, 2021, pp. 363–367). The building of the installation consisted of many different activities, such as typing words and painting wood. There is a direction and determinacy; as more of the installation is built, possible ways of constructing the installation make way for how the installation is actually built. Returning to Dewey’s formulation, where the situation forms the background for action, we can say that small-scale affordances allow for the anticipation of large-scale affordances. A situation brings its own large-scale affordance, and therefore, it is possible that the recognition of a situation allows us to anticipate certain actions. Thus, a situation inviting us to act on a large-scale affordance already diminishes the relevance of a number of small-scale affordances in the landscape of affordances.

## 4 Emotions as situational properties

People of similar capacity and skill may be in the same environment; they can find themselves in completely different situations. A certain setting or situation can be perfectly comfortable for men but very threatening for women. For instance, the Cairo public transportation system is dangerous to women, given the rampant sexual violence against women in those spaces (UN Woman, 2013; see also Crippen, 2021, 2022). A workplace is experienced differently by someone who is depressed than someone who is happy that they were recently promoted. The difference is not just a difference in subjective experience but an objective difference in how a space is arranged, what actions are possible, and the possible movements of interactions with others that determine the quality of experience. Gibson (1979, p. 132) gives us the example of the cliff face that affords falling and is therefore not just perceived as dangerous but is *objectively* dangerous.^8^ Our relation to the world is not one merely of skilled engagement but also depends on the affective mediation of one’s social position, (emotional) wellbeing, the overall quality of the space, and other non-skill-related factors. Gibson and neo-Gibsonian theories have only marginally addressed that our skilled engagement is an affective engagement—therefore, also emotional—and how affordances are effectively organized. I argue that when we consider these affective organizations through the lens of a situation, it becomes clear that not only do emotions influence how we perceive affordances, but that perceiving affordances means perceiving emotionally inflected spaces. Dewey emphasizes that a situation has a perceived pervasive qualitative character that gives the situation a qualitative wholeness (Dewey, 2008, p. 105). It is this pervasive quality that not only binds the constitutive elements of the situation into one whole but also individuates each situation into something unique (Dewey, 1986, p. 74).^9^ This means that we not only relate to situations in terms of perceived opportunities for action. However, we are also sensitive to the emotional and affective qualities of a situation. This, I argue, is central to our understanding of a person being open or closed to certain affordances, in the sense that the selective opening of an agent toward an affordance is not just the result of the agent’s skills and capacities but that spaces themselves can be open or closed, hostile or friendly, as an affordance.

Agents have to coordinate multiple affordances at the same time, and this requires a selective openness from the agent (Rietveld and Kiverstein, 2014; van Dijk and Rietveld, 2021), that is, not fully captured by the temporal unfolding of activities within a situation. The openness of an individual toward certain affordances (the solicitations of the affordance, the demand character, etc.) cannot be accounted for simply by the skills of the individual (cf., Dings, 2018; Withagen, 2022). There are differences in how people perceive affordances. It should, therefore, be noted that a situation pertains to more than action perception (Dreon, 2022, p. 60). We do not perceive the world simply in terms of what we can do, but we relate to the affordances in a particular way; they matter to us one way or another. All our interactions with the world are permeated by affectivity, as all interactions are mediated by the body. Colombetti (2014) argues that sensory experience, therefore, always has a necessary primordial affective aspect. Affectivity and emotion are not just possible elements of our skillful engagement or an in-between element of the perception-action loop but a pervasive and structural element of the mind (Colombetti, 2014, p. 63). Emotion affects how we perceive affordances. How steep we perceive a hill to be can depend on our general fitness and health, whether we are fatigued or encumbered, but it also depends on our mood (Riener et al., 2011). Depression saps our energy, making the hill less climbable. This means that perceiving emotional qualities is not just a matter of perceiving what people are feeling. Just like emotions prepare us for a certain action and draw our attention to certain elements of the situation, the perceived emotions in others also offer possibilities for action. Being angry inclines me to certain actions, but seeing someone who is angry, happy, sad, etc., affords me new actions. “[…] perceiving expressions is sometimes about reading minds, it is more squarely about perceiving solicitations or closures for action” (Crippen, 2021). This means that the anger of that person is not an internal state but one of the qualities of the interaction between us. For Dewey, emotional qualities are openings and closures, invitations to approach and avoid (2005, pp. 15–16). This means that the salience of affordances is affected by emotional interest. In addition, emotions also change the affordances themselves by restricting the possible range of behaviors through energy levels. Sadness closes certain affordances because with sadness comes weariness that changes our bodily dispositions and makes certain spaces less approachable and explorable as it makes the space feel exhaustive (Crippen, 2022). James (1985, p. 150) argued that it is the emotion that brings “value, interest, or meaning [in] our respective worlds.”^10^ According to Crippen, the close relation between interest and emotion, in that emotion is interest-like, is still largely missed in theory on cognition and emotion (Crippen, 2018, p. 342). For James, interest is selected by directing attention, creating bias, and thereby dividing reality into manageable bits. Interest and emotion make sure that we are not cognitively overloaded. Emotions motivate us; for instance, fear can motivate us to flee from a bear. Emotions also anticipate future situations, thereby orienting action. Fleeing from the bear also anticipates my future survival. Emotion has an object, and it is necessarily about something (Dewey, 2005, p. 67). Interest and emotion provide an interpretive and synthesizing framework through which we experience the world (Crippen and Schulkin, 2020, p. 49). As Gallagher (2020, p. 105) puts it: “In these pragmatic, affective, and hedonic embodied dimensions, saliency and meaning emerge.”

Crippen (2022) concludes, in line with Gibson, Merleau-Ponty, and Dewey, that environments have expressive properties. We perceive spaces as having physiognomic properties, as in we recognize the value of a thing immediately, just like the perception of color, and just like we recognize the emotions of a person in their face.^11^^,^^12^ Values are engendered in organism-environment relations, external to the perceiver (Gibson, 1979, p. 127). This also holds for the emotional qualities of things we encounter and, therefore, the emotional properties of situations. Although we often perceive emotions for mind-reading purposes, perceiving emotion has more to do with reading situations to deal with them. Emotions are generated in external interactions with things. Furthermore, these properties are not subjective projections but objective properties that operate as openings and closures. Environments are valuable to us in a particular way. For Crippen and Schulkin (2020, p. 46) and Crippen (2022), the emotional characteristics of the environment are very close to affordances. Environments can be exciting, gloomy, depressing, fun, romantic, upheaving, etc. These moods and emotions in the environments seem to be shared among individuals or simply perceived without having these moods and emotions yourself; they are more than projections of the mind unto the environment. We can perceive a party to be joyful, while we feel sad. In fact, the perceived joyfulness can be a reason to avoid the party. The perceived emotional quality of a threatening dark alleyway makes that a lone woman is in a different situation than, for instance, a group of professional boxers. Trails disappearing around a corner have an attractive mysteriousness and promise discoveries (Kaplan and Kaplan, 1989; Crippen and Schulkin, 2020; Crippen, 2022).

Emotions and moods delineate objective possibilities for action; in other words, they delineate worlds. Emotions push or pull us toward certain objects, activities, and interactions by making them salient and, therefore, guide action. Emotions are not something we project onto the world, as emotions introduce action-limiting constraints. Emotions are organizational patterns of the organism that, in relation to patterns in the environment, make certain affordances more relevant to us than others. Hence, a situation is an experienced environing world. It is, therefore, not only our skills that allow us to perceive possibilities for engagement but also our emotional resonance with the environment that makes us open to certain affordances and, therefore, open to employing certain skills. Emotions delineate the environing worlds by marking our interests. An alleyway is objectively dark and scary, which makes avoiding the alleyway or proceeding with caution a pertinent way of acting. It is a quality that, if we are aware enough of the situation, we can all perceive. We are attuned to patterns in the environment, thereby being shaped by the environment and, in turn, shaping the environment.

“The emotional worlds of the farmer, lawyer and grieving person accordingly vary in no small part because they come with different pushes and pulls. Yet their emotional worlds also overlap out of being pushed and pulled similarly, experiencing common threats and hopes and consequently needing to do similar things.” (Crippen, 2021)

The emotional attractions are critical for organizing sensorimotor exploratory activity into actions. Therefore, they also act to filter information relevant to the agent by shifting attention and action. The action shifts our attention to its contours, seeking to perpetuate itself, enriching the emotional appraisals of the situation, thereby tying activities and undergoing together in a qualitative meaningful whole, which is an environing world. These emotional pushes and pulls make certain activities and affordances relevant and organize action, allowing the situation to act as a large-scale affordance in itself. This can be positive and negative for the agent. Although experience is the result of skilled coping with the world, this coping is effectively mediated and directed. Emotions are orientations toward the world, a way of possible dealings with it. We perceive the world in terms of affordances because of our skillful engagement with the world, but we engage because we are pulled by the environment’s emotional qualities. Our relationship with the action space is, therefore, not separable from affectivity. In turn, affectivity is not separable from interaction. The environment reflects our likings, pulls us, pushes us, and brings about preferences, which are inseparable from the affordances that we perceive in the environment. It is the emotion that ties the situation together as a contextual whole by being its pervasive qualitative character.^13^ A situation is not merely a background for action but a background that has a certain qualitative character for us.

## 5 Places and territories

A situation brings about demands and opportunities for action, to which we can respond given that we have the right agential resources, i.e., skills and capacities, and environmental resources. As we are social creatures and our environment is fundamentally social, most of these resources are socially embedded. The same holds for our agential resources; we learn skills in a social setting and many through social interaction. The situation is primary in action, which means that, in effect, we learn how to respond to situations in a certain manner. Just like a closet does not afford dancing due to the space being too constricted for movement, so can social-cultural norms constrict action. We do not dance at funerals despite the space not being as constricting as a closet (Miyahara et al., 2021).^14^ The social norms governing our behavior at funerals, what we wear, etc., can be nearly as constricting as the walls of a closet, as breaking them can have significant social costs and, in certain cases, can produce genuine harm. What these norms are depends on the country or culture that we find ourselves in and other *situational* circumstances. The material features and social structures that we find ourselves in and that we grow up in have been structured by community members and those who came before us (Heft, 2018, p. 115). Changing cultural context changes the affordance; for instance, one can dance at an Irish or Hawaiian funeral. These changes can also be both material and social, such as the introduction of women-only carriages in the Cairo Metro, making the space accessible again to women. Artifacts and spaces do not only offer affordances because of their material properties but also because of the sociocultural coordination around the artifact or space.

Sociocultural coordination can offer new opportunities for action but can prevent people from acting on certain affordances. For certain groups, a specific social coordination can be dangerous or constraining. Affordances are, therefore, not just relational with respect to a person’s skills and capacities but also in relation to the social position a person inhabits. An entrance with only stairs is objectively unwelcome to people in a wheelchair. However, the same entrance can also lack lighting in the evening, which, combined with a culture that is harmful to certain people, can make the space genuinely threatening. The qualities of the space itself open or close certain affordances for us. Spaces are opportunities for movement and action, and we perceive these spaces emotionally. Dewey describes this qualitative character in relation to the loosening and thickening of space and time:

“Space is room, *Raum*, and room is roominess, a chance to be, live and move. The very word “breathing space” suggests the choking, the oppression that results when things are constricted. Anger appears to be a reaction in protest against fixed limitation of movement. Lack of room is denial of life, and openness of space is affirmation of its potentiality. Overcrowding, even when it does not impede life, is irritating. What is true of space is true of time. We need a “space of time” in which to accomplish anything significant.” (Dewey, 2005, p. 217)

Because we relate primarily to situations in regard to action and the situation acts as a large-scale affordance, we perceive spaces as affording us possible actions. The habitat of animals is full of places, such as places to hide and places to find food (Gibson, 1979, p. 34; Heft, 2018, p. 100). A space is, therefore, not a neutral background for agents but can be a *setting* for certain activities and a *place* to be in or belong to. What gives a place meaning is what the place affords. Because humans are fundamentally social creatures, they create and perceive emotionally inflected spaces (Crippen, 2022). Just like an angry face can solicit us to back off, so can a threatening space make us feel unwelcome. Social coordination works on an emotional level: “Emotionally hostile architecture features are far removed from a smile or outreached hand, being more akin to a pair of folded arms and other standoffish gestures that curtail opportunities for engagement” (Crippen, 2022). Thus, affordances can not only be social (e.g., van Dijk and Rietveld, 2017), but according to Crippen (2022), there are also political affordances, that is cultural spaces that have “affectively charged, non-subjective, normative openings and closures […] that give rise to selectively permeable barriers.”

A result of the material features and social structures, i.e., sociomaterial coordination, is the marking of spaces in the environment where certain actions, and hence certain forms of agency, can be performed. A classroom is a space clearly marked for learning if you are a student and marked for teaching if you are a teacher.^15^ The artifacts that are set up in the space provide affordances for action that are part of a certain learning process. However, there are also other markers, such as the habitual role that a teacher takes up, that we use to appraise the room. The lights, air ventilation, and ambient noises are all part of marking the space as a pleasant or annoying place to learn. We do not see the classroom as a space that affords learning but as well-suited or ill-suited for learning. The classroom, for someone passing by, can be nostalgic or a place of dread. The exact way the artifacts and the room itself are set up determines the way the learning situation unfolds. If the chairs and tables are directed toward the teacher, the classroom situation affords direct instructions from the teacher. If the tables are set up in groups toward each other, the classroom situation affords cooperative learning exercises. We are perpetually attuned to (common) dynamic patterns across types of places and relative differences among dynamic patterns between settings (Heft, 2018, p. 117). When growing up, it is not simply the case that we find that certain actions succeed in certain spaces, but our (burgeoning) actions are situated by our caretakers and other community members in certain places or as part of certain practices. Environmental patterns operate as markers because not only do we, as individuals, have a history of interaction with or within those spaces, but the spaces themselves have a history of interaction with other community members, present and past.

Many of these environmental patterns mark what can be called *territories* within an environmental space. Territory not only allows certain actions and, therefore, supports certain situations but also limits other actions or access to the place depending on the overall situation. An operating room is generally only accessible to a patient and medical staff. Depending on what role one has, your actions are quite limited. In other words, spaces can become territories because we relate to the environment via situations. These patterns can be explicit, such as a sign in a hallway that reads ‘No running’, but most of the time, these patterns operate on a tacit level. A classroom is a setting marked for certain learning activities. A home is marked as a setting to live in, making it friendly to those who live there. However, it is also marked as private, making it a hostile space to those who are unwelcome. These spaces can be modulated to a certain extent. Some modulations are intended, like the classroom, and some are not. An alleyway allows a person to walk home. However, when the alleyway has bad lighting at night, and there is a culture of harm toward women, this drastically changes the situation for a woman who seeks to make use of the space, even to the point where the situation can deny her agency with respect to the alleyway. For certain places, the possible kinds of interaction can be so limiting to specific agents that the place functions as having a selectively permeable barrier. Hence, we can speak of territory when the entrance to the space can effectively be denied to certain people or allow only a limited form of agency. A steep set of stairs might deny a person who is less mobile entrance to a building, but so can the requirement for formal attire for those who are not dressed or cannot dress appropriately, and the removal of benches at the train station can make a space hostile to homeless people. The environmental patterns of the alleyway are such that given the right organization of experiences of the agents, the alleyway is perceived as dangerous, foreboding, and something to be avoided. In other words, territories mark expectations for certain situations and allow us, therefore, to anticipate certain actions or weave the activities into overarching actions. Certain spaces, with their artifacts contained, offer a limited amount for the kind of situations it can be part of and anchor possibilities for certain kinds of interactions. A closely related idea is that of behavioral settings, originally proposed by Barker (1968) and further developed by Harry Heft (e.g., Heft, 2001). A behavior setting is a dynamic, quasi-stable pattern of behavior and milieu. A behavior setting can be described as a joint affordance that governs the behavior of multiple agents for some time in such a way that their behavior is interdependent. Examples are a language lesson in the aforementioned classroom, a game of chess, and a funeral. A behavior setting—although it must not be confused with the situation itself as one can join a behavior setting and one finds oneself in a situation—can be seen as a particular type of territory that appears at a certain location at a certain time due to a shared commitment to collective actions.

A territory is not just a place or set of places that affords certain actions but also selects for certain forms of agency. A territory is not only perceived in terms of what an agent can do but also in terms of what an agent cannot do. This can have the effect that a space can have a permeable barrier to those that cannot adhere to the expected form of agency. Either because they do not have the capacities or because their needs require a different form of agency. Therefore, a territory always holds a negative limiting affordance for certain agents. These territories are the result of previous and ongoing habitual adjustments of the environment. Note that it is not just the space itself but the transactional relationship between the environments and the agents that make up the situation. A home can be very friendly if I am invited in. I argue that through the notion of situation, agency is constituted or constrained via social transactions and the very spaces we move in because they have been molded by previous social transactions.

## 6 Four axes of analysis

Given the earlier considerations, I propose the following four axes of analysis for how action is constrained or solicited through situations. These are not meant to be independent of each other or to be fully exhaustive taken together. These four axes often intersect but are a way to analyze action and agency without giving primacy to one element in the organism-environment transaction of the situation.

The first axis is the *complexity* of a situation in relation to how the situation is spread out over the environment. Dewey (2013, p. 279) states that “a higher organism acts with reference to a spread-out environment as a single situation.” In spatial terms, a situation can be contained in a small locale, for instance, eating breakfast at home, or spread out over multiple environments like traveling by airplane. A situation can also be temporally spread out, such as a letter or email exchange. What is done in response to something nearby is tied to what is done in response to something far away. In a present action an extensive and during environment is implicated. More complex behavior works in two directions: first, the ability of the organism to relate to a more spread-out environment (or multiple environments) as a single situation. Second, the ability for an organism to identify more (relevant) elements within a situation for action. An example of the latter case would be the difference between someone who happens to drink wine and a wine connoisseur. It is the skills and previous experiences of the connoisseur that allow the person to distinguish more properties of the wine.

In the former case, a situation brings a serial time-spread order of separate events into a single unified whole to which a limited set of actions are relevant, therefore acting as a soliciting affordance. More complex organistic behavior is therefore an integration of different organism-environment interactions. The downside, which is not so explicit in Dewey’s work, is that a complex situation also means a more vulnerable context for action, as there are more elements that can disrupt the unfolding action. An organism is sensitive to a certain underlying infrastructure for action that serves as a large-scale affordance. In the case of taking a flight to a certain destination, this is a more typical understanding of infrastructure. The action would not be possible if there were no airports, buses, etc. Thus, through interventions in different environments, we can support actions that are spatially and temporally spread out. A situation is an extensive field of habits and affordances that is the result of an organism being reliant on a variety of environments. By relating to our interactions with these environments as situations, we enlarge our capacities to respond to challenges and possibilities for meeting our needs as organisms. Situation and action codefine each other. As actions flow into each other, so do situations. There can be a case that in order to compensate for the fragileness of the unfolding actions, intervening habits of others and/or infrastructure are required.

The second axis is the extent to which a situation is *determined*. In a fully determined situation, the flow of action is uninterrupted. However, life is full of interruptions and recoveries. In most cases, our habits are flexible enough, and we adjust our activities to keep the action unfolding. It is because we have to continuously adjust our working habits to the specific environmental circumstances that emotion and thought to arise (Dewey, 2007, p. 178). Normally, we remain sufficiently in harmony with our environment. A situation becomes problematic when actions and activities are blocked due to changing environmental circumstances and a situation is undetermined. Unanticipated elements or changes in the environment can block or disrupt crucial activities (Dewey, 2007, p. 51). Then, a process of inquiry follows, which does not necessarily mean a process of reflective deliberation. The agent is not sure how to proceed and must rely on habits of exploratory activity, such as reflection, in order to come to a better understanding of the situation. Dewey (1986, p. 108) defines inquiry as “the controlled or directed transformation of an indeterminate situation into one that is determinate in its constituent distinctions and relations as to convert the elements of the original situation into a unified whole.” The constituent parts of an indeterminate situation do not hang together. The situation is too open and disjointed for the agent to make sense of what to do. Inquiry is controlled or directed because, if done competently, it can bring about a unified situation. A fully determined situation is a situation that is finished and not open anymore for any further action. A situation that is unfamiliar or contains unfamiliar relevant elements to us is problematic, as we have not settled on a response to the demands of the situation. Arriving at the airport, while waiting for our flight, we find out that our flight is canceled. Instead of getting a coffee and reading a book or mindlessly scrolling on the phone, we must actively look for a solution. That is, finding an alternative mode of travel, taking a different plane, or even canceling the trip means changing the situation.

The third axis is the *(social) establishment* of a situation and refers to the (social) expectations and anticipations within a situation. Bruner (1990) stated that in an established situation, we have no need to understand each other by means of belief/desire schema unless the actions deviate from what falls within the range of our anticipations. As discussed earlier, when it comes to social situations, not only do our own emotions make objects and activities more salient by making certain affordances more relevant, but so do the perceived emotions of others and the space we find ourselves in. We often have to adjust our actions if the perceived emotions and spaces do not match up in relation to our understanding of the situation we find ourselves in. Objects stand out in relation to the pervasive qualitative character of the situation. Thus, when we perceive the emotions of others, in general, our attention is drawn to those aspects of the situation that are relevant to them, forcing us to adjust our activities and come to a better understanding of the situation. If a situation is well-established, we are familiar with what is expected of us as agents. When we enter a party, and contrary to expectations, the mood is dim; it is the emotional patterns of the other people involved in the situation that solicit us to change action: inquire what is wrong. A similar thing holds for unfamiliar spaces. That is, we have a hard time identifying what is relevant for a smooth traversal of the space. This can also happen if too many new elements are introduced in familiar social territories, and this can occur outside direct dyadic interactions.

The fourth and final manner of analyzing the situation concerns the *restrictiveness* of a situation. The restrictiveness does not pertain to the flow of action but to the number of activities that are available to the agent at a certain point in the situation. Unlike when a situation is undetermined, whereby the possible action becomes unclear when a situation is restrictive, it constrains the possible number of activities in such a way that the action becomes overdetermined. Action becomes overmechanical and, therefore, more fragile to changing environmental circumstances. A situation must always constrain action simply by introducing relations of relevancy. A situation that does not constrain activities is problematic, as no force guides an organism to certain activities. In that sense, an unconstrained situation is not a situation. However, when a situation becomes too restrictive, a change in the environment can more easily stop an unfolding activity, and because the organism has no other available activities at hand, the change derails the action. Thus, it could be argued that when a situation is too uniform, it is directly constraining activities, or when succeeding situations are too similar in properties with respect to the agent, there is no longer any substantive agentive activity at play. Habits have become mere repetitions, taking away any possibility for growth for the organism. A stereotypical example of this would be working in a factory line, where one deviant activity can derail the entire (joint) action and problematize the situation, as the agent cannot perform an alternative activity. A situation can be restrictive because both physical characteristics, such as the confines of a closet, do not afford dancing, and sociocultural characteristics, such as dancing at a funeral, go against cultural norms.

## 7 Conclusion

I perceive a chair as having one specific and relevant affordance because I see the chair and myself as part of a situation. Situations come about by means of the organism organizing its experience of the environment as a conceptual whole. This is not an internal process. As social creatures, we collectively organize situations by habitually shaping the world and each other by making certain affordances more relevant in relation to the field of affordances. This implies that our perception of the spaces surrounding us is not separable from affectivity. Interventions in spaces shape situations in such a manner that certain forms of agency are made possible or are denied, which can result in spaces being closed off to certain persons.

A situation is an environing experienced world. It is the background for action to unfold that has a unity (that speaks of a world). What determines the range of a situation is its *relevance* to the activity or actions of the agent. A situation is the integration of affect in perception such that the situation has a pervasive qualitative character that gives the situation contextual wholeness. We do not only perceive emotions contextually, but (especially social) situations have an emotional quality that pulls us toward or drives us away from certain actions. A situation acts like a large-scale affordance because it effectively organizes the affordances it contains in terms of relevancy and availability. The dynamics of concern, ability, and the possibilities of territories make up a situation as affordance and determine the demand character of smaller-scale affordances. How a situation shapes action can be analyzed along four axes: complexity, determinedness, establishment, and restrictiveness.

## Data availability statement

The original contributions presented in the study are included in the article/supplementary material, further inquiries can be directed to the corresponding author.

## Author contributions

TH: Writing – original draft, Writing – review & editing.

## Footnotes

^1^Often simply equated with context.

^2^Despite many positions in philosophy and the cognitive sciences acknowledging the importance of situations, there is a significant lack of elucidation on what a situation is in the literature, with some notable exceptions [for instance, in the study by Heft (2001, 2018)]. There is no standard account of situation. For instance, a strong theoretical perspective on situations is lacking in the field of social psychology (Yang et al., 2009). However, many of the themes discussed can be found, for instance, those working in the tradition of phenomenology or philosophical anthropology.

^3^Gibson (1982, p. 410) states that he distinguishes affordance from the positive or negative valences of things since the former are objective properties of the environment and the latter change when the internal state of the observer changes. However, in certain places in his study, Gibson can be interpreted as being more ambiguous as he equates affordances to non-subjective values. For instance, when he talks about the difference in odor, that is, the prey being different than that of a predator’s odor, the one being negative and the other being positive (Gibson, 1979, p. 137; see also Crippen, 2020). For the purpose of this study, the problem that affordances sometimes solicit action and sometimes not, and the problem that affordances are experienced differently remain underdeveloped in Gibson’s work. Because a chair affords sitting, it on occasion, also solicits sitting. Whether an affordance invites or solicits action seems more subject-dependent as the soliciting effect requires an observer, while an affordance does not require an observer (Withagen et al., 2012, p. 256; Dings, 2018, p. 684).

^4^Please note that Gallagher’s intent is to answer the question: ‘how do we individuate action?’ Although closely related to this study, I will not fully explore this question here. What suffices is that the example shows that action cannot be understood in isolation of its context.

^5^In this study, I do not adhere to the common belief/desire model of agency.

^6^A point that is often misunderstood in Dewey, even by experts (see for instance Burke, 1994, p. 22), is that situations not only arise when a harmonious interaction between organism and environment breaks down, i.e., a problematic situation. However, we are always part of a situation, and I further develop his account of situational dynamics.

^7^Note that the problem of relevance of affordances was already a prime concern in the study by Rietveld and Kiverstein (2014).

^8^Another good example is Achmed’s (2006) discussion of Husserl’s table. The table must have the appropriate properties for Husserl to work on it, but also be placed in a particular room in his summer house, and kept clean by the unseen labor of others.

^9^Dewey speaks of tertiary qualities which are not about certain parts of the situation, and not to be confused with primary and secondary qualities such as mass and color. They are experienced immediate and pervade all elements and relations. Hence, it ties different experiences in a single situation, until its completion, and we move into a different situation. Dewey compares tertiary qualities to the esthetic appreciation of a painting such as a painting having a Titian or Rembrand quality (Dewey, 1986, p. 75).

^10^James’ notion of selective interest is therefore an affective notion.

^11^Although physiognomic qualities were traditionally applied to faces, it was the Gestalt psychologists who applied the term to environmental objects. Gibson (1979, p. 138) addresses physiognomic qualities in his discussion of Gestalt Psychology, and in particular Kurt Kofka (1935), in relation to the origin of the concept of affordance. Crippen (2021) expands on their understanding by arguing that we read human faces and spaces the same way.

^12^Not only do emotions influence how we perceive the situations, but Crippen (2021) has argued that also our perception of emotions is situationally dependent. According to Crippen, the Kuleshov effect, wherein identical shots of performers manifest different expressions in different contexts, shows that the identification of emotion is contextually dependent. We read faces like we read situations. That is, we perceive the person’s expression of the emotion in his contextual whole.

^13^The emotion in question can be (and very often is) ambivalent or even change. For instance, anger turning into happiness or sadness. However, we do not experience ambivalence or changing of emotions as a series of well-defined loose experiences. It is a single experience.

^14^At least in the culture the author of this study is embedded.

^15^Note that the classroom given the right joint actions and practices counts as a behavior setting as defined by Heft (2001, 2018).
